# Empyema as an Unusual Presentation of Lung Cancer

**DOI:** 10.7759/cureus.96674

**Published:** 2025-11-12

**Authors:** Faisal Arslan, Joohi Khan, Arsh Dhupar, Navneet Kaur, Kamran Amin, Ali Hussain

**Affiliations:** 1 Cardiology, Pinderfields Hospital, Wakefield, GBR; 2 Geriatrics, Pinderfields Hospital, Wakefield, GBR; 3 Acute Medicine, Pinderfields Hospital, Wakefield, GBR; 4 General Medicine, Pinderfields Hospital, Wakefield, GBR; 5 Endocrinology, Pinderfields Hospital, Wakefield, GBR

**Keywords:** chest tube, hypoxemia respiratory failure, lung cancer, pulmonary empyema, thoracentesis, thoracocentesis

## Abstract

Malignant empyema is a rare presentation of lung cancer, marked by the accumulation of frank pus in the pleural space. It can result from various mechanisms, including direct tumor invasion, airway obstruction, immunosuppression, and secondary infections. The clinical presentation is often complex, with symptoms overlapping those of the underlying malignancy and potential complications like sepsis. In the present case, the patient initially presented with chest infection symptoms but, on further investigations, was diagnosed with lung malignancy. Treatment requires a multidisciplinary approach focused on draining the infected fluid, managing infection with antibiotics, and addressing the underlying cancer. Overall, the prognosis of malignant empyema is poor and often fatal.

## Introduction

Malignant empyema refers to a collection of pus in the pleural space that occurs in association with cancer, most commonly lung cancer or metastatic cancer to the pleura. It is a life-threatening diagnosis and requires prompt diagnosis and treatment [1]. However, it is uncommon; less than 0.3% of lung cancer patients present with an empyema, making it a rare complication [2]. The diagnosis is often challenging and difficult to make because of overlapping symptoms it shares with underlying malignancies and infections. There could be several etiologies for empyema in cancer patients, which could be direct sequelae of cancer per se or as a result of chemotherapy, radiotherapy, or invasive procedures [3]. Pleural fluid should be analyzed for the possibility of both malignancy and underlying infectious etiology, such as pneumonia or serious infections like tuberculosis. Once a diagnosis is made, a pleural biopsy can aid in diagnosing cancer cell type, and a positron emission tomography (PET) scan is prudent to know the extent of disease, which will help in further planning management [2].

Despite advancements in medical interventions, the treatment of malignant empyema remains challenging. The prognosis for patients with malignant empyema is generally poor, reflecting the severe nature of this condition and its impact on an already vulnerable patient population.

## Case presentation

**Figure 1:**
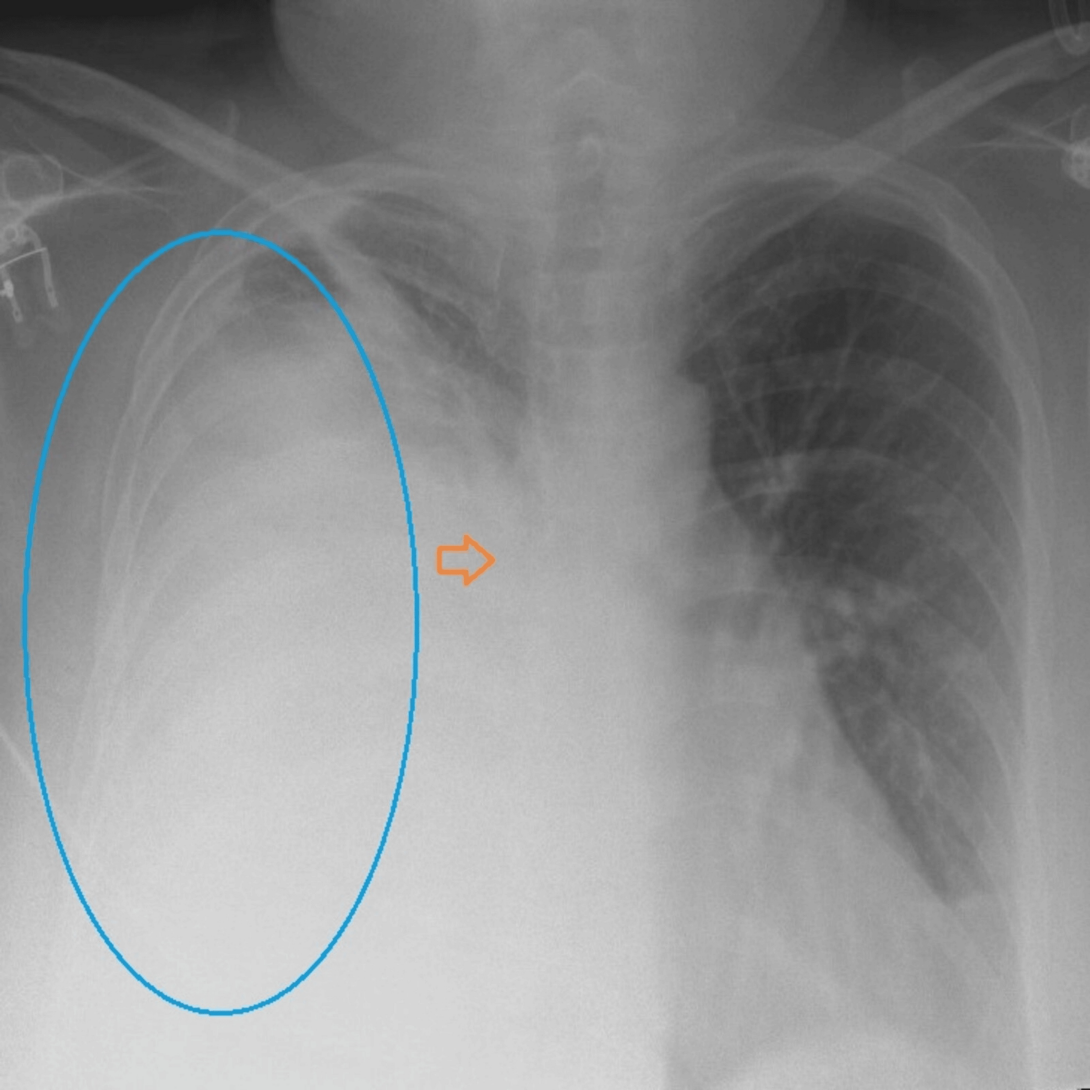
The chest X-ray shows a large right-sided white-out of the lung (shown in blue marking) with a loss of bronchial markings (orange arrow), indicating compression.

A 55-year-old female presented to the emergency department with a five-day history of worsening breathlessness and right-sided chest pain. Her symptoms began six months ago with a productive cough that occasionally had streaks of blood. There was no medical history of constitutional symptoms, such as low-grade fever, weight loss, changes in appetite, or night-time sweating. She initially consulted her primary care physician, who prescribed antibiotics for a presumed chest infection. She was a chronic smoker (one pack per year) but had quit six months prior. She had no history of industrial dust exposure or previous respiratory diseases. Upon initial assessment in the emergency department, she was afebrile but found to be tachypneic, with a respiratory rate (RR) of 35 breaths per minute, tachycardic with a heart rate of 130 beats per minute, and hypoxic with an oxygen saturation (SaO_2_) of 70% on room air. She was started on oxygen via a 15 L non-rebreathing mask. Laboratory investigations revealed a raised C-reactive protein (CRP) level of 502 mg/L (range: 0-9 mg/L) with a white blood cell count of 26.0×10^9^/L (range: 4.0-11.0×10^9^/L) and predominant neutrophils of 21.95×10^9^/L (range: 2.5-7×10^9^/L). A chest X-ray showed a large right-sided white-out of the lung with a meniscus sign and loss of the right-sided tracheal lining, indicating its compression (Figure 1).

**Figure 2:**
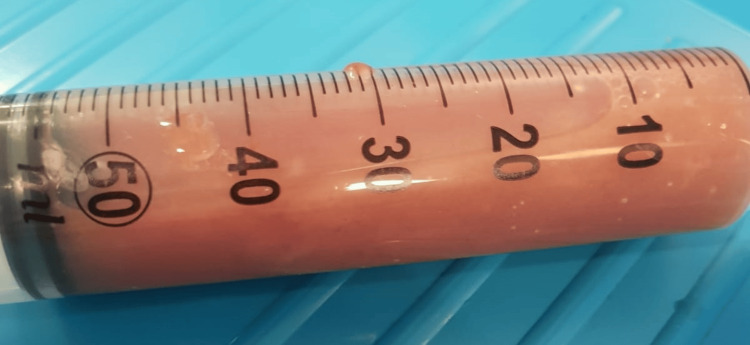
Frank pus from chest.

Bedside chest ultrasound confirmed the presence of a complex, heterogeneous effusion. Upon needle insertion, thick, foul-smelling frank pus was aspirated with a pH of 7 (Figure 2). An 18 French intercostal chest drain was inserted under ultrasound guidance using the Seldinger technique to prevent blockage in the emergency department. Within an hour of insertion, 800 mL of frank pus was drained. The patient was also started on intravenous (I.V.) antibiotics, levofloxacin 500 mg twice daily, and metronidazole 500 mg three times daily.

Cytological examination of the pleural fluid revealed a high concentration of pus cells on Gram stain, although no organisms were identified in cultures over one week. Pleural fluid smears for acid-fast bacilli were negative, and tuberculosis cultures remained negative over six weeks. Pleural fluid analysis revealed a protein level of 42 g/L (normal range: <30 g/L) and an LDH level of 1,800 U/L (normal range ≈ 200 U/L).

Following clinical improvement, the drain was removed after five days, and intravenous antibiotics were converted to oral therapy - levofloxacin 500 mg twice daily and metronidazole 400 mg three times daily - for a planned duration of four weeks, as advised by microbiology. Follow-up was arranged in the pleural disease clinic.

**Figure 3:**
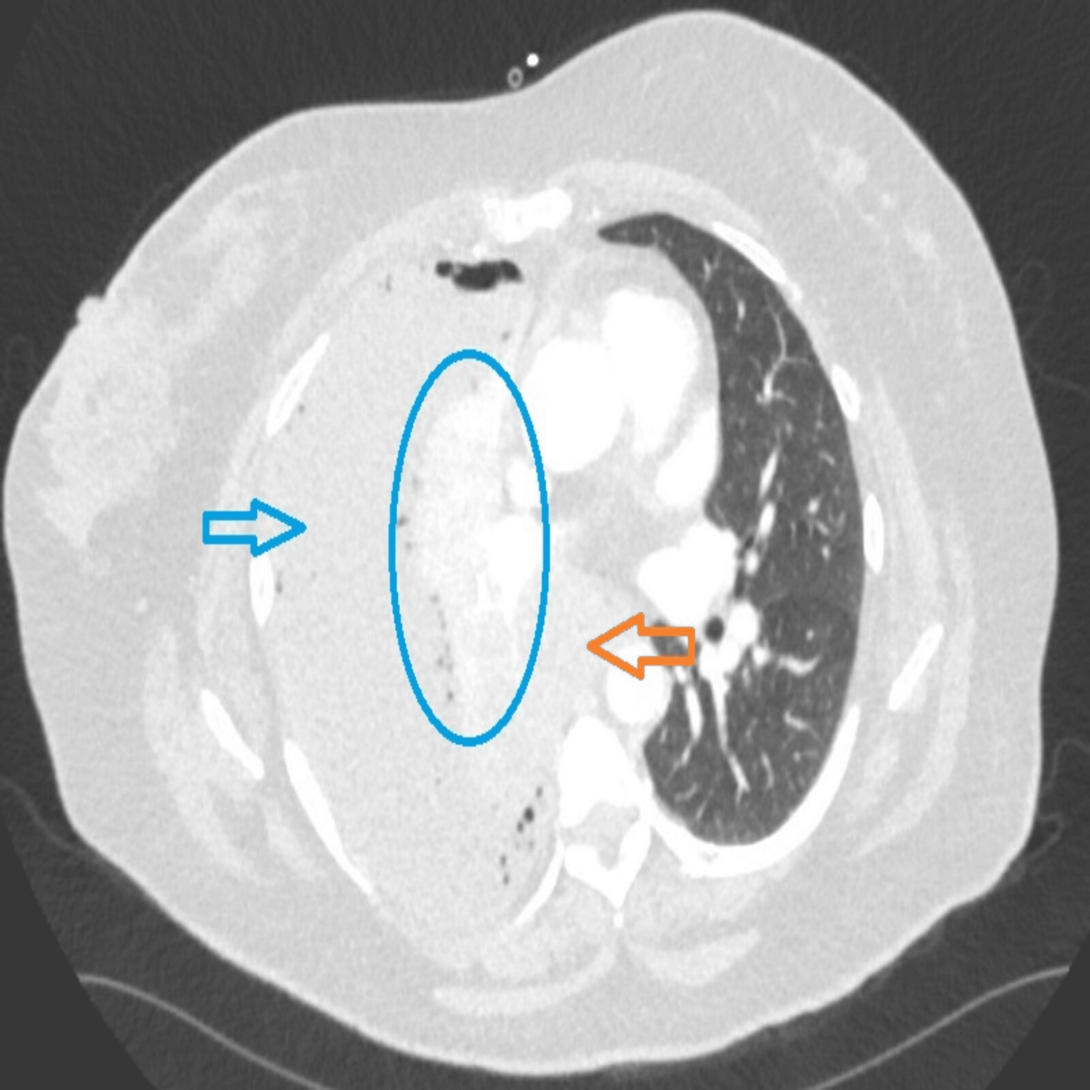
CT chest shows large empyema. The blue arrow indicates empyema, the collapsed lung is shown by the blue circle, and paratracheal invasion is indicated by the orange arrow.

However, the patient was readmitted with worsening shortness of breath. A CT chest scan revealed marked enlargement of the right hilar mass with invasion into the mediastinum and suspicion of an enlarging left paratracheal lymph node (Figure 3). Following a multidisciplinary team (MDT) discussion, bronchoscopy was performed, and tissue samples were taken. Histopathology and immunohistochemistry findings were consistent with squamous cell carcinoma. Tumor cells exhibit PD-L1 expression in approximately 60% of cases. Molecular testing reveals negative results for EGFR, KRAS, BRAF, ALK, MET exon 14 skipping, ROS1, RET, and NTRK1-3 alterations. Therefore, a diagnosis of squamous cell carcinoma in situ was made.

**Figure 4:**
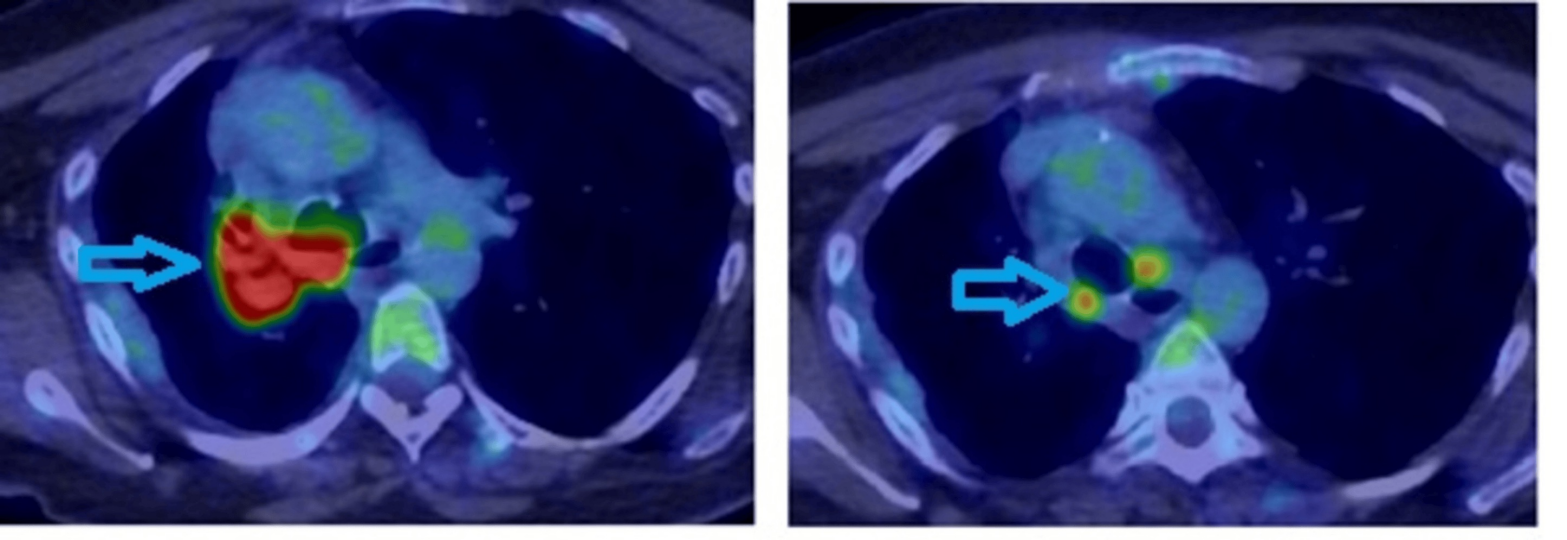
PET scan showing large mass and peri-tracheal lymph nodes uptake (arrows).

A subsequent PET scan demonstrated an intensely hypermetabolic right hilar mass measuring 75×65 mm, occluding the right main bronchus (Figure 4). Radiological staging of the lung mass showed metabolic appearances consistent with locally advanced right hilar non-small cell lung cancer (NSCLC), with a metabolic stage of T4 N3 M0/1a.

Due to the patient's poor functional status and the size and distribution of the disease, she was offered high-dose palliative therapy. She underwent high-dose palliative radiotherapy to the right chest, which was completed in 13 fractions. Despite treatment, further investigations six months later indicated disease progression. A multidisciplinary team meeting concluded that transitioning the patient to best supportive care was the most appropriate course of action.

## Discussion

Thoracic empyema is an infectious process defined by frank pus in the pleural space. An empyema due to malignancy is a collection of pus in the pleural space (the area between the lungs and chest wall) that occurs as a complication of cancer [4]. This condition is also known as malignant empyema or cancer-associated empyema. The common causes are direct extension of tumor, obstruction of the airways or lymphatics, immunosuppression, esophageal perforation, bronchopleural fistula, hematogenous spread, invasive procedures, or idiopathic etiology [5]. It is important to note that while empyema can occur in cancer patients due to these mechanisms, not all empyema in cancer patients is directly caused by malignancy. Sometimes, it may be due to secondary infections unrelated to the cancer.

The clinical manifestation of malignant empyema can be challenging to interpret, as its symptoms often resemble those of the primary cancer and other associated complications. Symptoms like persistent fever, pleuritic chest pain, dyspnea, productive cough, fatigue, weight loss, and night sweats are commonly seen [4]. Other relevant findings on examination may suggest that tachypnea, tachycardia, decreased breath sounds on the affected side, and, rarely, clubbing may also be observed.

Depending on the primary cancer site, patients may present with additional symptoms such as hemoptysis (in lung cancer), dysphagia (in esophageal cancer), or lymphadenopathy. Signs of sepsis may be present in severe cases, including altered mental status, hypotension, and organ dysfunction [6]. It is important to note that the clinical presentation may be atypical or subtle in some cancer patients, especially those who are elderly or immunocompromised.

The strata of patients who are at risk of developing empyema are those with an already underlying malignancy or metastasis or those undergoing chemotherapy leading to immunosuppression. Patients with a compromised immune system due to HIV, long-term steroid use, smoking, swallowing difficulties, and prone to aspiration pose a high risk of developing empyema [7]. Comorbid conditions like chronic obstructive pulmonary disease (COPD), diabetes mellitus, and liver cirrhosis also add an iota of risk, on top of being in an older age group.

Diagnosing this condition involves clinical assessment, imaging studies, and laboratory tests. Clinically, patients may present with symptoms, as discussed above. On imaging, a chest X-ray is usually the first imaging performed, as it can show fluid accumulation in the pleural space and is also helpful in looking for any obvious abnormalities in the chest. It often presents unilaterally with noticeable asymmetry, causing blunting of the costophrenic angle. Even smaller effusions can be identified using a lateral X-ray. Sometimes, decubitus views help evaluate layering and better quantify the effusion. Furthermore, it can also aid in diagnosing a lung mass. Ultrasound is used to detect and characterize fluid, guide thoracocentesis, and assess for locations. A CT scan of the chest provides detailed imaging of the chest, helping identify the extent of empyema, any underlying pathology, or potential malignancies [8]. PET-CT may be used to detect and stage malignancies.

Thoracentesis is a procedure that involves inserting a needle into the pleural space to obtain a fluid sample. The fluid is analyzed for appearance (purulent in empyema), cell count and differential, biochemical markers (pH, glucose, LDH, protein), microbiological culture, and sensitivity and cytology to look for malignant cells [9]. A pleural biopsy can be done if malignancy is suspected but not confirmed by fluid cytology. This can be done via CT-guided needle biopsy or thoracoscopy. A routine complete blood count (CBC), C-reactive protein (CRP), and erythrocyte sedimentation rate (ESR) should be performed, in addition to blood cultures, to aid in treatment. Depending on the suspected type of cancer, specific tumor markers may be tested in the blood or pleural fluid. Bronchoscopy should be considered if there is suspicion of an endobronchial lesion causing the empyema. Molecular and genetic testing may also be done later to guide treatment decisions. The diagnostic approach may vary depending on the patient's clinical presentation and the healthcare setting. The goal is to confirm the presence of empyema, identify the underlying malignancy if not already known, and guide appropriate treatment [10].

The treatment approaches for empyema due to malignancy depend upon several factors and often require a multidisciplinary approach. The primary goal is to remove the infected fluid from the pleural space. Thoracentesis, chest tube insertion, and video-assisted thoracoscopic surgery (VATS) can all be done for drainage.

Broad-spectrum antibiotics are typically started empirically. The choice of antibiotics may be adjusted based on culture results and antibiotic sensitivity testing. Antibiotic therapy typically lasts 2-6 weeks, depending on the clinical response [4,11]. Agents like tissue plasminogen activator (tPA) and deoxyribonuclease (DNase) may break down loculations and improve drainage. Management of underlying malignancy is crucial for long-term control and may include chemotherapy, radiation therapy, immunotherapy, or targeted therapies [12]. Pleurodesis may be considered to prevent recurrence of pleural effusions. It involves inducing adhesion between the visceral and parietal pleura using talc, bleomycin, or tetracycline. Nutritional support and adequate pain management are vital for patient comfort and to facilitate effective breathing and coughing [13]. Oxygen therapy or mechanical ventilation may also be necessary in severe cases.

Depending on the management plans, regular monitoring with imaging studies and clinical assessments is crucial to ensure resolution and detect any recurrence. However, in advanced malignancy cases, the focus may shift from symptom management to improving quality of life. It is important to note that treatment should be tailored to the individual patient, considering factors such as the type and stage of malignancy, overall health status, and patient preferences. The management of empyema due to malignancy often requires balancing the treatment of the infection and the management of the underlying cancer.

The prognosis for patients with empyema due to malignancy is generally poor. This is because it represents a severe complication in patients who are already dealing with cancer. The median survival time can range from a few months to about a year, depending on various factors. The development of complications such as sepsis, respiratory failure, or bronchopleural fistula can significantly worsen the prognosis. Some patients may not be candidates for aggressive treatments due to their overall condition, which limits treatment options and affects outcomes.

## Conclusions

Empyema due to malignancy represents a severe and complex challenge in cancer care. The condition necessitates careful diagnostic and therapeutic strategies to balance the immediate need for infection control with long-term cancer management. Timely and accurate diagnosis is essential due to the complexity of symptoms and potential complications. Although multifaceted and intensive treatment often provides limited improvement in prognosis, highlighting the importance of personalized care plans and the need for ongoing research into better management practices. Future research should focus on palliative care strategies aimed at improving the quality of life for affected patients.
